# Problematic Social Media Usage and Anxiety Among University Students During the COVID-19 Pandemic: The Mediating Role of Psychological Capital and the Moderating Role of Academic Burnout

**DOI:** 10.3389/fpsyg.2021.612007

**Published:** 2021-02-05

**Authors:** Yan Jiang

**Affiliations:** ^1^School of Humanities, Tongji University, Shanghai, China; ^2^Psychological Counseling Center, Shanghai University, Shanghai, China

**Keywords:** problematic social media usage, anxiety, academic burnout, epidemic, psychological capital, COVID-19, university students

## Abstract

The outbreak of COVID-19 has greatly affected university students’ studies and life. This study aimed to examine the possible mediating role of psychological capital and the moderating role of academic burnout in the relationship between problematic social media usage and anxiety among university students during COVID-19. A total of 3,123 undergraduates from universities in Shanghai participated in an online survey from March to April 2020. The results showed that problematic social media usage among university students predicted their levels of anxiety. Mediation analysis indicated that psychological capital mediated the relationship between problematic social media usage and anxiety. Furthermore, for university students whose academic performance had been affected by the COVID-19 pandemic, the effects of both problematic social media usage and the psychological capital on anxiety were moderated by academic burnout. For university students whose academic performance was not affected by the COVID-19 pandemic, academic burnout moderated the effects of psychological capital but not the effects of problematic social media usage on anxiety. The results highlighted the underlying mechanisms in the relationship between problematic social media usage and anxiety. These findings provide practical insights into the development and implementation of psychological interventions when facing a pandemic.

## Introduction

The COVID-19 pandemic that began in December 2019 has spread to more than 200 countries and regions and infected 79,094,442 patients globally (96,074 in China) as of 24 December 2020. The pandemic brought not only the threat of physical health but also psychological stress, such as anxiety, depression, and fear among the public (Song, 2020). Previous studies have demonstrated the negative effects of infectious outbreaks and subsequent quarantine orders on both posttraumatic stress disturbance (Hawryluck et al., 2004) and psychological stress (Lau et al., 2005; Brooks et al., 2020) experienced in the general population. Given the wide social impact of the pandemic and the governmental response, including physical distancing measures and quarantine, the COVID-19 pandemic might have psychiatric consequences. The results from a recent meta-analysis documented high levels of both posttraumatic (26.2%) and psychological (23.1%) stress associated with COVID-19 (Cooke et al., 2020). Furthermore, patients diagnosed with COVID-19 in the isolation ward and/or with general pneumonia in the observation ward had different degrees of anxiety, depression, and sleep problems (Yang et al., 2020). Sizable proportions of the public not infected with COVID-19 reported panic and anxiety (Islam et al., 2020; Wang et al., 2020). For both the infected and non-infected population, anxiety may be particularly prevalent and devastating during this pandemic due to uncertainty, reduction in economic income, and the accompanying lack of a sense of security.

As a result of the COVID-19 pandemic, the learning and lifestyle of university students in China have undergone drastic changes. To curb the spread of the pandemic, universities delayed the spring semester and students were, in advance, asked not to return to university and stay at home as much as possible. Consequently, long periods of isolation at home and uncertainty about when to return to university may increase the risk of anxiety among university students (Wang et al., 2020). Additionally, universities have started using internet platforms to develop different types of online courses, for example, requiring students to complete their study tasks at home via social media. Although previous studies reported a positive influence of social media usage on mental health and well-being (Clark et al., 2018; Glaser et al., 2018), many studies have found that the excessive use of social media has negative effects on users (Steers et al., 2014; Muench et al., 2015; Nesi and Prinstein, 2015; Hormes, 2016). During the pandemic, university students who were forced to stay at home had to learn, communicate, and obtain the latest information about the pandemic from social media, thus increasing the time and frequency of mobile social media usage. Given that preoccupation and the excessive amount of time spent on social media are symptoms of problematic use, excessive social media use among Chinese university students may easily turn into problematic use (Andreassen et al., 2012). Therefore, the present study focused on university students’ problematic social media usage during the pandemic. Overuse and problematic social media usage have been linked to poor psychological well-being (Huang, 2017) and symptoms of depression (Twenge et al., 2018), and anxiety (Vannucci et al., 2017; Elhai et al., 2020). Therefore, changes in the learning style, lifestyle, and problematic social media usage have become possible risk factors for university students’ anxiety.

Thus far, the extent to which the COVID-19 pandemic and problematic social media usage affect the levels of anxiety among university students in this context have not been empirically addressed. To fill this gap, this study recruited students from universities in Shanghai as participants. After the COVID-19 pandemic was effectively controlled in China (from the end of March to the beginning of April 2020), an online questionnaire was distributed to systematically investigate the relationship between problematic social media usage and anxiety. Concurrently, this study focused on the mediating role of psychological capital between levels of anxiety and problematic social media usage, as well as the moderating effect of university students’ academic burnout on the effect of problematic social media usage on anxiety and related behavioral mechanisms. Although prior studies had found a close relationship between problematic social media usage, anxiety, and psychological capital, the pattern of the relationships may be changed during COVID-19. In the following paragraphs, I introduce the theoretical background and then propose the hypotheses.

### Theoretical Framework and Hypotheses Development

#### The Relationship Between Problematic Social Media Usage and Anxiety Among University Students During the COVID-19 Pandemic

Anxiety refers to an unpleasant sense of fear and apprehension, which is characterized by uneasiness derived from anticipating danger, the unknown, or unrecognized (Allen et al., 1995). The COVID-19 pandemic is an example of such a situation, in which the outbreak is sudden and highly infectious, and the current knowledge or treatment of the disease is limited, which can have devastating effects on the mental health of people and produce anxiety (Ho et al., 2020). For university students, the outbreak has had two main effects on their studies and life. First, university campuses were closed, thus students could not attend classes or continue to complete research in the laboratory as usual, which might have disrupted their original study plans and added more uncertainty to their future academic development (Liu et al., 2020). In addition, with the introduction of long-term home quarantine, most university students and their families had to study or work at home while living together in a confined space. This scenario could lead to an increase in the likelihood of family conflict and, thereby, increase individual anxiety levels (Chen et al., 2020; Wu et al., 2020). Therefore, the academic performance of some students, which refers to “how students deal with their studies and how they cope with or accomplish different tasks given to them by their teachers” (Masrek and Zainol, 2015, p. 3,604), will be affected by the pandemic. Recently, the breakdown from the Office for National Statistics (2020) showed that young adults’ anxieties during the pandemic often revolved around the effect on their university and the impact on the quality of education in the United Kingdom. Similarly, research by Cao et al. (2020) found that higher levels of anxiety were associated with factors strongly related to COVID-19 among Chinese medical university students. For these reasons, university students may be particularly vulnerable to anxiety during the pandemic. Based on the above literature review, Hypothesis 1 is proposed:

Hypothesis 1 (H1):The levels of anxiety will increase among the university students whose academic performance has been affected by the COVID-19 pandemic.

The second major impact among university students was from the problematic social media usage, which refers to being preoccupated with social media, having a strong motivation to use social media, and spending too much time on social media, which leads to impairments in their social, personal, and/or professional life, as well as psychological health and well-being (Andreassen and Pallesen, 2014). Most universities have developed online courses to deal with the impact of the COVID-19 pandemic on the curriculum plans. The use of social media has been forced to be prolonged, which may lead to the emergence of problematic use of social media, and thus hurt university students’ mental health. Previous studies have shown that excessive use of social media can increases anxiety (Lepp et al., 2014). Especially during the pandemic, people who frequently use social media may receive a lot of negative information, even fake news, related to the situation, which may, in turn, increase the levels of anxiety (Gao et al., 2020). Moreover, the internet platform has become the only way to attend classes, receive notices from universities, and attend meetings, so the passive use of social media, such as browsing content, has increased among university students. Thorisdottir et al. (2019) investigated social media use and symptoms of anxiety among 263 Icelandic adolescents and suggested that passive use of social media was related to greater anxiety symptoms for both genders. Both active and passive excessive use of social media by university students is likely to develop into problematic use of social media. Previous studies have demonstrated a close relationship between problematic social media usage and anxiety (Hussain and Griffiths, 2018; Wong et al., 2020). Against this background, I propose the following hypothesis:

Hypothesis 2 (H2): During the pandemic, problematic social media usage predicts the anxiety levels of university students.

#### The Mediating Effect of Psychological Capital

Psychological capital refers to an individual’s positive psychological state, which consists of four psychological resources: self-efficacy, optimism, hope, and resilience (Luthans et al., 2007a). According to key resource theories, these resources, and thus psychological capital, can help individuals successfully cope with, alleviate, or eliminate the negative effects of stress and maintain mental health (Thoits, 1994; Bandura, 1997; Wright et al., 1998; Hobfoll, 2002). Several studies have revealed that psychological capital is an important psychological mechanism relating stress and health. For example, Fang et al. (2014) found that negative life events decreased mental health by reducing psychological capital among university students. Furthermore, Yang (2016) suggested that an increase in stressful events leads to a decrease in individuals’ well-being, and psychological capital plays a mediating role between stressful events and well-being. In the context of COVID-19, Mubarak et al. (2020) suggested that psychological capital effectively alleviated individuals’ fear. Therefore, psychological capital may be an important psychological mechanism for university students to deal with COVID-19 pandemic and maintain their mental health.

The growth of psychological capital depends on the development of positive resources and finding new ways to deal with psychological problems (Simsek and Sali, 2014). However, excessive social media usage or smartphone addiction is regarded as an escape behavior (Kwon et al., 2011), which is harmful to cultivating psychological capital. Specifically, excessive internet usage would provide a virtual world where individuals seem to control everything and generate a virtual state of strength that does not enable them to cope with problems in reality (Boellstorff, 2008; Simsek and Sali, 2014). Furthermore, spending much time and energy on the internet reduces the investment of time and energy in cultivating psychological capital (Simsek and Sali, 2014). Some studies also suggested that excessive internet or smartphone usage was closely correlated with psychological capital. For example, Zhang and Jin (2015) found that smartphone addiction negatively correlated with psychological capital and Simsek and Sali (2014) revealed that internet-addicted students were more likely to have lower psychological capital. Furthermore, many university students who were asked to complete study tasks through mobile phones increased their time and frequency of mobile social media usage during COVID-19 (Dong et al., 2020; Prakash and Yadav, 2020), which enhances the chances of problematic use of mobile social media, giving rise to various negative effects (Duan et al., 2020; Elhai et al., 2020). Thus, I speculate that problematic social media usage may impede the development of psychological capital and propose the following hypothesis:

Hypothesis 3 (H3): During the pandemic, problematic social media usage can significantly negatively predict individuals’ psychological capital.

Psychological capital is also significantly correlated with anxiety (Dian et al., 2020). According to the ego depletion theory (Muraven and Baumeister, 2000), psychological capital comprised of positive psychological resources may be limited. If individuals lack psychological capital, they cannot effectively cope with stressful events and suffer from negative emotions, such as anxiety and depression (Baumeister et al., 1998; Rahimnia et al., 2013). In other words, individuals with high psychological capital have more positive resources to cope with stress, thereby reducing anxiety, whereas individuals with low psychological capital have fewer positive resources to cope with stress, thereby increasing anxiety. Many empirical studies have verified that psychological capital is beneficial in dealing with stress and eliminating anxiety. For example, Wu et al. (2019) found that an increase in psychological capital effectively alleviated university students’ anxiety. Liu et al. (2013) revealed that psychological capital could help reduce anxiety symptoms among employees diagnosed with HIV/AIDS. Dian et al. (2020) found that high psychological capital, especially self-efficacy, and optimism reduced the levels of anxiety for market fire victims. Moreover, it has also been shown that enhancing youth’s psychological capital could reduce their social anxiety during the COVID-19 pandemic outbreak (Li, 2020). Therefore, it is conceivable that psychological capital would negatively predict anxiety among university students.

Based on the literature review above, problematic social media usage negatively predicts individuals’ psychological capital, and decreases in psychological capital can exacerbate anxiety. Therefore, the following hypothesis is proposed:

Hypothesis 4 (H4): Psychological capital plays a mediating role in the relationship between problematic social media usage and individuals’ anxiety.

#### The Moderating Effect of Academic Burnout

In addition to the negative effect of problematic social media usage on anxiety and the mediation role of psychological capital, another concern is that during the COVID-19 pandemic, the effect of the problematic social media usage on anxiety will be stronger. In particular, the learning-related variables would interact with problematic social media usage, thus jointly influencing the general anxiety of university students during the pandemic. In this study, given its close connection with psychological capital and anxiety, I focused on the role of academic burnout, a common negative psychological trait among university students, in the relationship between problematic social media usage and anxiety, as well as its mechanism.

Academic burnout was defined as a negative psychological syndrome caused by long-term, excessive academic pressure. Facing academic burnout, students gradually lose energy and have reduced enthusiasm for learning, a lower sense of achievement, and a lack of positive attitudes owing to long-term academic burden (Meier, 1983). Researchers have suggested that academic burnout consists of three dimensions: emotional exhaustion, cynicism, and a low sense of accomplishment (Lee and Ashforth, 1996; Houkes et al., 2011). Some researchers have found that burnout may be a significant moderator, and different levels of burnout tend to have beneficial or detrimental effects on a series of outcomes (Baba et al., 2009; Akanni et al., 2019). For instance, individuals with higher burnout levels are less likely to be engaged in their job, even though their emotional intelligence is high (Akanni et al., 2019). According to Kao and Chang (2017), the interaction effect of burnout and job stress can affect the well-being of individuals. Additionally, emotional exhaustion, a dimension of burnout, plays a moderating role in the relationship between proactive personality and individual performance. That is, individuals with less proactive personalities performed worse under circumstances of high levels of emotional exhaustion (Baba et al., 2009). Therefore, it is reasonable to consider academic burnout as a moderating variable in this study.

In addition, during the outbreak of the COVID-19 pandemic, universities closed their doors and students had to attend classes and take examinations by using social media, which increased their levels of academic burnout (Zis et al., 2020). Learning online poses a great challenge to students, which can easily result in excessive usage of social media. Moreover, social media overuse may easily turn into problematic social media usage, which can result in emotional problems for people (Yildiz Durak and Seferoǧlu, 2019). In the case of the COVID-19 pandemic, it remains unclear what effects learning-related variables may have on the relationship between problematic social media usage and general anxiety. Besides, according to the model of job resources-demand, similarly, academic burnout is the result of the constant consumption of psychological resources (Demerouti et al., 2001). In this case, when university students with a high level of academic burnout are faced with academic stress, the association between problematic social media usage and anxiety may be increased, owing to individuals’ lack of psychological resources. Although psychological capital acted as a moderator in previous studies (Shaheen et al., 2016; Guo et al., 2018), I mainly focus on the moderating role of the learning-related variable in the current research, and academic burnout can be considered as the consumption of psychological resources. Thus, academic burnout, instead of psychological capital, may act as a moderator in my research.

More specifically, it has been shown that anxiety is more likely to be the result of academic burnout (Chang et al., 2012). This means that the higher the levels of burnout, the higher the levels of anxiety. According to Han’s (2017), students with high academic burnout were more likely to be dependent on social media, possibly to avoid studying. Additionally, the overuse of social media was linked to students’ anxiety (Woods and Scott, 2016); thus, I proposed that students with high academic burnout levels would strengthen the relationship between problematic social media usage and students’ anxiety.

Furthermore, in terms of the relationship between burnout and psychological capital, most researchers agreed that the higher the level of psychological capital, the lower the level of burnout (Wang et al., 2012; Laschinger and Fida, 2014; Estiri et al., 2016). This is because psychological capital, as a kind of positive mental status, affects the ways students respond to difficulties. To be more accurate, students with higher levels of psychological capital are less likely to have symptoms of academic burnout. Simultaneously, as mentioned above, students’ anxiety also increased with an increase in burnout (Chang et al., 2012).

In general, according to Pluess (2015), the interaction between the environment and the individual has different developmental effects based on their sensitivity to the environment. That is, if one is sensitive to changes in the environment, they may experience extreme emotions. Social media usage is deemed an environmental factor, and psychological capital and academic burnout are deemed psychological factors. The interactions among these factors can influence individuals in different ways. Previous research has found that excessive mobile social media usage may result in anxiety (Woods and Scott, 2016); therefore, when academic burnout, as a coordinator, interacts with other factors, it also influences students’ anxiety. In addition, in the context of the pandemic, academic burnout may interact with the pandemic and, thus, affect students’ anxiety. Students who are affected by the pandemic are more sensitive to environmental changes and more prone to academic burnout. Therefore, the moderating effect of academic burnout may be different among students based on whether their academic performance is affected by the pandemic or not. Consequently, the following hypothesis is proposed.

Hypothesis 5 (H5): Academic burnout moderates the relationship between problematic social media usage and students’ anxiety (H5a) and between psychological capital and students’ anxiety (H5b), and moderated effects are different among students based on whether their academic performance is affected by the pandemic or not.

### The Present Study

**FIGURE 1:**
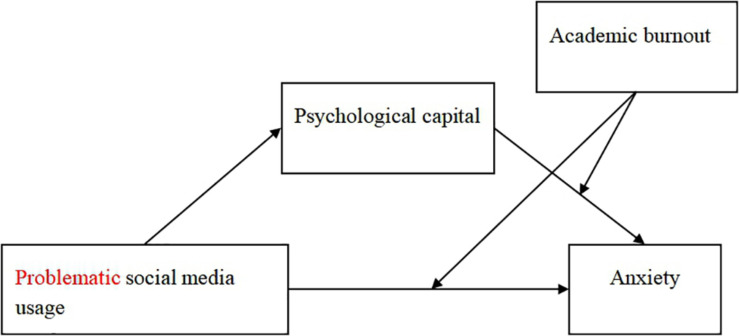
The proposed moderated mediation model.

In this study, Chinese university students were recruited as participants, and an online questionnaire was administered during the COVID-19 pandemic. It is well known that COVID-19’s outbreak at the beginning of 2020 changed people’s life. To avoid the further spread of the pandemic, the Chinese Ministry of Education postponed the start of school and notified students about the need to complete their studies in the form of online classes at home. This form is very different from traditional learning methods and is an academic challenge for many students. Some students could not adapt to this methodology. The lack of group discussions and the weakening of the interactive learning environment greatly reduced their learning efficiency and enthusiasm. Therefore, subjectively, they believed that the pandemic negatively affected their academic performance. However, other students who had orderly planned their studies felt that the changes in the external environment and learning methods could not hinder their learning process and academic enthusiasm. They were in the same state as before. These students did not believe that the pandemic affected their academic performance. Only those students who subjectively believed that they were affected by the pandemic were truly victims of the pandemic. The pandemic did not affect the academic performance or mental health of students who considered themselves unaffected. Given the different subjective feelings of individuals, the pandemic had different effects on the psychological structure of individuals and these effects should be discussed separately. Therefore, I divided the students into two groups according to their subjective perception and explored the psychological structure of the two groups of students to reveal the more comprehensive impact of the pandemic. In the actual investigation, the students needed to answer a question about whether the pandemic affected their academic performance. According to their answers, they were divided into two groups: university students who reported that they were affected by the pandemic (Group 1) and those who reported not being affected by the pandemic (Group 2). First, I compared the differences in problematic social media usage, psychological capital, anxiety, and academic burnout between the two groups. Then, I integrated problematic social media usage, anxiety, psychological capital, and academic burnout into a model to examine the mediating and moderating mechanisms underlying the relationship between problematic social media usage and anxiety. Figure 1 illustrates the proposed model.

## Materials and Methods

### Participants

The questionnaire was distributed online during the spring semester of 2020. A total of 3,123 undergraduates from universities in Shanghai participated in the survey. To distinguish between students whose academic performance was affected by the pandemic and students whose academic performance was not affected, I included the following item: “Whether COVID-19 has affected your academic performance or not.” The responses were coded as: “yes” or “no”. Then, I recoded the responses into two categories: 1 (affected) and 2 (not affected). According to participants’ self-reports, 2,056 students (816 males, 1,240 females) believed that their academic performance was affected by the COVID-19 pandemic (Group 1) and 1,067 (739 males, 328 females) students believed that their academic performance was not affected by the COVID-19 pandemic (Group 2).

### Measures

#### Anxiety

Anxiety was measured using the GAD (Generalized Anxiety Disorder, GAD-7) self-report scale (Spitzer et al., 2006). Qu and Sheng (2015) tested the Chinese version of the scale and proved its reliability and validity. The scale consisted of seven items (e.g., “Over the past 2 weeks, have you often worried too much about different things?”). Each item was rated on a 4-point Likert-type scale, ranging from 0 (not at all) to 3 (nearly every day). The scale had no reverse scoring, and all items were added to obtain a total score. The higher the total score, the more serious the anxiety symptoms. Cronbach’s α coefficient was 0.93 in the current sample.

#### Psychological Capital

The Positive Psychological Capital Questionnaire was used to assess participants’ psychological capital (Zhang et al., 2010). The scale contains 26 items, each assessed using a 7-point scale that ranges from 1 (very inconsistent) to 7 (very consistent). Items such as “I have great confidence in my ability” were included in the scale. Five items required reverse scoring before calculating the total score. After reverse scoring the negative items and summing the item results, a higher score indicated that individuals have more psychological capital. The Cronbach’s α coefficient for the scale was 0.93 in the current study.

#### Problematic Social Media Usage

The problematic social media usage of the participants was assessed using the Problematic Mobile Social Media Usage Assessment Questionnaire (Jiang, 2018). The questionnaire consisted of 20 items (e.g., “I have a certain dependence on mobile social networks, and sometimes I cannot control my playing time.”) mainly related to the duration, frequency, and intensity of mobile social media use and the negative impact of excessive use on the individual’s physiology, psychology, and cognition. Each item was rated on a 5-point Likert-type scale, ranging from 1 (totally inconsistent) to 5 (totally consistent). The scale had no reverse scoring. Summing the items, higher scores indicate greater problematic social media use. In the current study, Cronbach’s α coefficient was 0.93.

#### Academic Burnout

Academic burnout levels were measured using the Maslach Burnout Inventory-Student Survey (MBI-SS) (Schaufeli et al., 2002). The Chinese version was revised by Fang et al. (2009) and had good reliability and validity. The scale had 15 items measuring three aspects of academic burnout: exhaustion, cynicism, and efficacy related to academic work. A sample item was “I feel burned out from my studies.” Each item was assessed using a 7-point Likert-type scale, with responses ranging from 1 (very inconsistent) to 7 (very consistent). After reverse scoring six items, all the items were added to calculate the total score. A high score indicated high academic burnout, and the Cronbach’s α coefficient was 0.90.

### Data Analysis

First, to examine the difference between Groups 1 and 2, an independent sample *t*-test was computed using SPSS 25.0. A *P*-value of less than 0.05 was considered statistically significant. Second, I calculated the correlation between the four variables of the two groups. Third, regression analysis was used to examine the relationships between problematic social media usage and anxiety and test hypothesis 2. Then, to examine hypotheses 3 and 4, the PROCESS macro (Model 4), which provides indirect effects of problematic social media usage on anxiety with 95% bias-corrected confidence intervals obtained from 1,000 bootstrap resamples, was used to test the mediation effect of psychological capital. The mediation effect was significant when the 95% confidence interval did not include zero. Finally, I used the PROCESS (Model 15) to test the moderated mediation effect, which allowed us to investigate whether the mediation process was moderated by academic burnout. The moderation effect was regarded as significant if the bootstrapped 95% confidence interval did not contain zero. If the moderation effect was significant, a simple slope analysis was also conducted using the PROCESS macro to further explore the nature of the moderation effect. This allowed us to verify hypotheses 5a and 5b. In addition, a previous study revealed that gender might affect anxiety (Xu et al., 2012). Therefore, in all analyses, participants’ gender was included as a covariate.

## Results

### Descriptive Statistics and Correlation Analysis

The descriptive statistics and correlations for all measures are presented in Table 1. According to the *t*-test, students of Group 1 and Group 2 differed significantly in terms of their problematic social media usage, psychological capital, academic burnout, and anxiety. These results supported hypothesis 1, indicating that the levels of anxiety increased among the university students who believed that their academic performance had been affected by the COVID-19 pandemic.

**TABLE 1:** Means, standard deviations, bivariate correlations, and *t*-test of variables.

Variables	*M*	*SD*	1	2	3	4	*t*
(1) Anxiety	5.46 (3.94)	4.28 (3.91)	–				9.95***
(2) Problematic social media usage	59.74 (53.57)	14.51 (15.38)	0.39** (0.32**)	–			11.05***
(3) Psychological capital	124.73 (129.33)	20.02 (21.54)	−0.43** (−0.45**)	−0.30** (−0.33**)	–		−5.93***
(4) Academic burnout	44.62 (40.03)	12.93 (13.41)	0.44** (0.42**)	0.40** (0.39**)	−0.65** (−0.71**)	–	9.18***

*p < 0.05, **p < 0.01, ***p < 0.001, all tests were two-tailed. The data outside the brackets were for university students whose academic performance had been affected by the pandemic (n_1_ = 2056), and the data inside the brackets were for university students whose academic performance had not been affected by the pandemic (n_2_ = 1067).

Based on the correlation test, problematic social media usage had a significant positive correlation with anxiety in both groups. For these students, psychological capital was significantly negatively correlated with anxiety and problematic social media usage. Academic burnout had a significantly positive correlation with anxiety and problematic social media usage and a significantly negative correlation with the psychological capital of students.

### Problematic Social Media Usage and Anxiety

I used linear regression to test hypothesis 2. The results showed that problematic social media usage was positively associated with the anxiety level of participants in Group 1 (*b* = 0.39, *SE* = 0.02, *p* < 0.001) and Group 2 (*b* = 0.32, *SE* = 0.03, *p* < 0.001). This means that regardless of what the students reported on their academic performance, problematic social media usage predicted university students’ anxiety levels. Therefore, hypothesis 2 was supported.

### Mediation Analysis

To examine hypotheses 3 and 4, a mediation analysis was conducted on both groups using PROCESS (Model 4). Among university students whose academic performance had been affected by the COVID-19 pandemic, there was a significant negative effect of problematic social media usage on psychological capital (*b* = −0.30, *SE* = 0.02, *p* < 0.001) and of psychological capital on anxiety (*b* = −0.35, *SE* = 0.02, *p* < 0.001). The indirect effect of psychological capital on the relationship between problematic social media usage and anxiety was significant (*b* = 0.10, *SE* = 0.01, 95% CI = [0.082, 0.125]). In addition, the residual direct effect of problematic social media usage on anxiety was also significant (*b* = 0.29, *SE* = 0.02, *p* < 0.001) (see Table 2).

**TABLE 2:** Testing the mediation effect of psychological capital.

Predictor	Model 1			Model 2		
	Psychological capital			Anxiety		
	*b*	*se*	*t*	*b*	*se*	*t*
Gender	0.11 (0.11)	0.04 (0.06)	2.66** (1.79)	0.07 (0.03)	0.04 (0.06)	1.73 (0.46)
Problematic social media usage	−0.30 (−0.33)	0.02 (0.03)	−14.03*** (−11.34***)	0.29 (0.19)	0.02 (0.03)	14.58*** (6.61***)
Psychological capital				−0.35 (−0.39)	0.02 (0.03)	−17.57*** (−13.76***)
R^2^	0.09 (0.11)			0.26 (0.24)		
F	106.08*** (68.11***)			246.22*** (109.88***)		

Gender was dummy coded such that 1 = male and 0 = female. Model 1 regressed psychological capital on problematic social media usage and gender; Model 2 regressed anxiety on problematic social media usage, psychological capital, and gender, respectively; the b values were standardized coefficients. The data outside the brackets were for university students whose academic performance had been affected by the pandemic (n_1_ = 2056), and the data inside the brackets were for university students whose academic performance had not been affected by the pandemic (n_2_ = 1067). *p < 0.05, **p < 0.01, ***p < 0.001.

Similarly, among university students whose academic performance was not affected by the COVID-19 pandemic, there was also a significant negative effect of problematic social media usage on psychological capital (*b* = −0.33, *SE* = 0.03, *p* < 0.001) and of psychological capital on anxiety (*b* = −0.39, *SE* = 0.03, *p* < 0.001). The indirect effect of problematic social media usage on anxiety via psychological capital was also significant (*b* = 0.13, *SE* = 0.02, 95% CI = [0.092, 0.170]). In addition, the direct relationship between problematic social media usage and anxiety was also significant (*b* = 0.19, *SE* = 0.03, *p* < 0.001) (see Table 3). Thus, irrespective of whether students’ academic performance was affected by the pandemic, psychological capital played a mediating role between problematic social media usage and anxiety.

**TABLE 3:** Testing the moderated mediation effect of problematic social media usage on anxiety.

Predictor	Model 1			Model 2		
	Psychological capital			Anxiety		
	*b*	*se*	*t*	*b*	*se*	*t*
Gender	0.11 (0.11)	0.04 (0.06)	2.66** (1.78)	0.03 (0.01)	0.04 (0.06)	0.81 (0.80)
Problematic social media usage	−0.30 (−0.33)	0.02 (0.03)	−14.03*** (−11.34***)	0.26 (0.17)	0.02 (0.03)	12.60*** (5.91***)
Academic burnout				0.17 (0.13)	0.03 (0.04)	6.58*** (3.38***)
Problematic social media usage × Academic burnout				0.08 (0.04)	0.02 (0.02)	4.32*** (1.53)
Psychological capital				−0.25 (−0.30)	0.02 (0.04)	−10.16*** (−7.87***)
Psychological capital × Academic burnout				−0.06 (−0.06)	0.02 (0.02)	−3.78*** (−2.57*)
R^2^	0.09 (0.11)			0.30 (0.26)		
F	106.08*** (68.11***)			146.99*** (61.27***)		

Gender was dummy coded such that 1 = male and 0 = female. Model 1 regressed psychological capital on problematic social media usage and gender; Model 2 regressed anxiety on problematic social media usage, academic burnout, problematic social media usage × academic burnout, psychological capital, psychological capital, academic burnout, and gender, respectively; the b values are standardized coefficients. The data outside the brackets were for university students whose academic performance had been affected by the pandemic (n_1_ = 2056), and the data inside the brackets were for university students whose academic performance had been not affected by the pandemic (n_2_ = 1067). *p < 0.05, **p < 0.01, ***p < 0.001.

### Moderation Effects

I used the PROCESS macro (Model 15) by Hayes (2017) to examine the moderating effects of academic burnout on the path from problematic social media usage to anxiety as well as the path from psychological capital to anxiety. A moderated mediation analysis was conducted on the two groups of participants. The results are summarized in Table 3.

For university students whose academic performance had been affected by the COVID-19 pandemic, problematic social media usage positively predicted anxiety, which was moderated by academic burnout (*b* = 0.08, *SE* = 0.02, *p* < 0.001). I further tested the interaction effect at high and low levels of academic burnout (i.e., one standard deviation above and below the mean of academic burnout) to investigate the moderating effects. The results showed that the association between problematic social media usage and anxiety was stronger for participants with a high level of academic burnout (*b*_*simple*_ = 0.35, *SE* = 0.03, *p* < 0.001) compared to those with a low level (*b*_*simple*_ = 0.17, *SE* = 0.03, *p* < 0.001). In addition, university students’ psychological capital negatively predicted anxiety, and these effects were moderated by academic burnout (*b* = −0.06, *SE* = 0.02, *p* < 0.001). The simple tests showed that the relationship between psychological capital and anxiety was stronger for participants with high levels of academic burnout (*b*_*simple*_ = −0.33, *SE* = 0.03, *p* < 0.001) than for those with low levels of academic burnout (*b*_*simple*_ = −0.19, *SE* = 0.03, *p* < 0.001).

For university students whose academic performance was not affected by the COVID-19 pandemic, the relationship between problematic social media usage and anxiety was not moderated by academic burnout (*b* = 0.03, *SE* = 0.02, *p* = 0.13). However, the relationship between psychological capital and anxiety was moderated by academic burnout (*b* = −0.06, *SE* = 0.02, *p* < 0.05). The simple tests showed that the relationship between psychological capital and anxiety was stronger for participants with high levels of academic burnout (*b*_*simple*_ = −0.37, *SE* = 0.04, *p* < 0.001) compared to those with low levels of academic burnout (*b*_*simple*_ = −0.25, *SE* = 0.04, *p* < 0.001). The above results suggest that hypothesis 5 was partially supported.

## Discussion

The present study investigated the relationship between problematic social media usage and anxiety among university students during the COVID-19 pandemic. It also aimed to test a moderated mediation model in which the effects of problematic social media usage on anxiety were contingent on the intervening processes of psychological capital and academic burnout.

The results suggested that levels of anxiety were significantly higher among university students who perceived their academic performance to be affected by the COVID-19 pandemic compared to those who did not share this perception. Previous studies have also shown that pandemics of severe infectious diseases present the loss of resources, uncontrollable stress, and a crisis in mental and psychosocial health (Leone et al., 2014). As a recurring feature of human history, large-scale pandemics of emerging infectious diseases likewise presents not only the challenges of the disease itself but also the risk of anxiety symptoms. This is consistent with a previous study, which indicated that the population became more pessimistic after experiencing the pandemic of the severe acute respiratory syndrome (SARS) (Neuner et al., 2006). Except for the anxiety caused by the COVID-19 virus itself, the pandemic has disrupted university students’ lives and study schedules, which may have further increased their anxiety.

Another lifestyle change triggered by the pandemic that may increase anxiety levels among university students is the use of mobile social media. In the present study, I found that problematic social media usage during the COVID-19 pandemic was significantly associated with anxiety among university students. Prior research also revealed that adults who put more effort and time into mobile social media had poorer sleep quality, lower self-esteem, and higher levels of anxiety and depression (Vannucci et al., 2017). These findings can be explained by the displacement hypothesis, which states that the use of the internet, especially to connect with people online, displaces face-to-face social relationships and the quality of social support given and received. These aspects negatively impact an individual’s mental health (Huang, 2010). Moreover, during public health crises, mobile social media spreads disinformation, thus aggravating public fear and panic. The prevalence of the pandemic has forced university students’ social capital to turn toward online connections. During the pandemic, mobile social media has played a pivotal role in the learning, living, and leisure activities of university students. Due to the long hours at home, e-learning has become the primary mode of learning for university students, whereas face-to-face social relationships have been kept to a minimum, both of which have created conditions and opportunities for social media usage and resulted in a significant increase in the amount of time university students spend on social media. Overreliance on social media certainly leads to higher anxiety among university students. A recent study also supported that during the COVID-19 pandemic, young adults who used social media frequently reported greater symptoms of depression and loneliness (Lee et al., 2020).

In addition, my study found that problematic social media usage affected the levels of anxiety through the mediation of psychological capital during the pandemic. Specifically, when university students use more social media, they typically have lower levels of psychological capital and, thus, higher levels of anxiety. These results are consistent with a recent study, which found that psychological capital had a mediation effect between coping style and anxiety among university students (Wu et al., 2019). An increase in internet and social media use time increases the possibility of internet addiction. Previous studies have also shown that individuals using the internet for social communication were more likely to be addicted to the internet than those who used the Internet for entertainment/news (Simsek and Sali, 2014). However, excessive internet use presupposes individuals to adopt negative coping styles of escapism, resulting in a sense of hopelessness and loss of control in life, which reduces psychological capital (Fincham et al., 2009). More specifically, the concept of psychological capital emphasizes the development of positive attributes to cope with psychological problems, which requires individuals to solve problems, rather than escape them. As a developmental structure, psychological capital requires an investment of time and energy (Fincham et al., 2009). For university students, spending too much time and energy on social media may deplete the internal mechanisms needed to cope with challenges and maintain a higher level of psychological capital.

Moreover, the negative predictive effect of psychological capital on mental health has been demonstrated in previous studies across cultures and populations (Krasikova et al., 2015; Tan, 2016; Xiong et al., 2020). Rahimnia et al. (2013) found that high psychological capital reduced destructive emotions, such as stress and anxiety, and eventually increased well-being. Specifically, when individuals have high psychological capital, they are equipped with extra resources to cope with stress, expect good things to happen, quickly “bounce back” after setbacks, and are more hopeful about negative situations (Shen et al., 2014). Therefore, university students with high psychological capital might have some control over their lives and have positive psychological capacities and motivation to cope with obstacles (Luthans et al., 2007b), which partly counteracts the effects of negative life events and stressors on mental health and reduces anxiety and depression. In sum, psychological capital played a mediating role in the relationship between social media usage and anxiety among university students.

This study also assessed whether university students perceived that their academic performance was affected by the pandemic. As mentioned above, I believe that for the students who perceived their academic performance to be affected, there were interactions between the pandemic and variables such as individual mobile media usage. However, the relationship between problematic media usage and other psychological constructs in the group who did not perceive any significant change in their academic performance was stable and less affected by the pandemic. By comparing the different patterns of relationships between these variables in the two groups, I hope to shed some light on the individual-specific effects of the pandemic. In the current study, I found that for the students who did not perceive their academic performance to be affected by the pandemic, the effects of problematic media use on anxiety and the mediation of psychological capital were also significant, similar to those who perceived their academic performance to be affected by the pandemic. This may be because for students who perceived their academic performance to be affected by COVID-19, the effect of problematic mobile social media usage on anxiety through psychological capital was the reflection of the external environment and a product of special situations (First et al., 2020; Lin et al., 2020). However, students who perceived their academic performance not to be affected by the pandemic but used mobile media more frequently, were not immune to the negative effects of overuse of social media. These students were likely to have already problems with excessive usage of mobile social media as a substitute for real-life face-to-face social networking in their daily lives (Glaser et al., 2018). In other words, these students were already at risk for fragile relationship networks, low offline social capital, and high internet addiction in non-pandemic periods, while being minimally affected by the pandemic. Moreover, for university students who perceived that they were not affected by the pandemic, psychological capital also had a strong role in mediating the relationship between problematic social media usage and anxiety.

Finally, the present study found that academic burnout moderated two pathways among university students: the influence of problematic social media usage on anxiety and that of psychological capital on anxiety. That is, problematic social media usage was more predictive of anxiety among university students with high levels of academic burnout than those with low levels. Specifically, students with high academic burnout feel exhausted because of study demands, have a detached attitude toward schoolwork, and have lower self-efficacy as a student (Zhang et al., 2007; Rahmati, 2015). In this case, social media overuse is more likely to be an approach to escape academic tasks, which increases the risk of internet addiction, resulting in higher levels of anxiety (Fincham et al., 2009). For students with low academic burnout, the sensitivity of the relationship between problematic social media usage and mental health may be relatively weak. Thus, students with low academic burnout were less affected by the influence of problematic social media usage on anxiety. Moreover, academic burnout also moderated the effects of psychological capital on anxiety. Students with high academic burnout similarly strengthened the relationship between psychological capital and anxiety. High academic burnout leads to university students being in a state of exhaustion, cynicism, and low efficacy, which brings to the fore the negative impact of low psychological capital on mental health outcomes (Schaufeli et al., 2002). In contrast, when individuals experience low academic burnout, the protective role of psychological capital in reducing anxiety may not be sufficiently realized (Shen et al., 2014).

Interestingly, the current study also found that academic burnout moderates different patterns of the relationship between problematic social media usage, psychological capital, and anxiety in the two groups of students. Our results demonstrated that for university students who perceived their academic performance to be unaffected by the pandemic, academic burnout only moderated the effects of psychological capital on anxiety but not the effects of problematic social media usage on anxiety. This could be interpreted considering another finding of this study, that is, the students who perceived their academic performance to be unaffected by the pandemic had lower levels of academic burnout compared to the other group, which is also consistent with the study of Peinado and Anderson (2020). Thus, the moderating role of academic burnout was also relatively weak in the former group of students. Furthermore, for the university students who perceived their academic performance to be affected by the pandemic, their problematic mobile social media usage was largely influenced by external stressors, such as COVID-19 exposure, fear of COVID-19, and COVID-19 misunderstanding (First et al., 2020; Lin et al., 2020). Conversely, for those who thought that their academic performance was unaffected by the pandemic, the high frequency and problematic mobile social media usage was attributed to preexisting factors, such as their personality (Correa et al., 2010), self-esteem (Bányai et al., 2017), attachment style (Blackwell et al., 2017), and a variety of other factors. Thus, the role of problematic social media usage on anxiety was more stable, leading to a more limited and insignificant moderating effect of academic burnout.

### Limitations and Implications

The present findings have both theoretical and practical implications. First, to the authors’ knowledge, this is the first systematic exploration of the relationships between problematic social media usage, psychological capital, anxiety, and academic burnout among university students during the COVID-19 pandemic. The findings of this study enhance the understanding of how and when problematic social media usage affects anxiety among university students. They further show that psychological capital was an important psychological mechanism among university students and enrich the research related to psychological capital and anxiety among university students. Second, the findings provided a new perspective on how to alleviate university students’ anxiety determined by the use of mobile social media. Furthermore, the mediating role of psychological capital and the moderating role of academic burnout suggest that I may be able to alleviate anxiety by improving students’ psychological capital or reducing their academic burnout so that these can counteract the negative effects of problematic media usage.

However, the study has some limitations. First, this study adopted a self-report method, in which participants reported whether they were affected by the pandemic. It is worth noting that these effects may be influenced by other factors, such as personality. Therefore, further research should collect data from multiple perspectives to reveal the relationships more accurately among these variables in different backgrounds. Second, I used a cross-sectional design to explore the mechanism of problematic social media usage on university students’ anxiety. Further studies should use a longitudinal-based design to establish causal relationships between problematic social media usage, psychological capital, academic burnout, and anxiety. Finally, prior research has revealed that the negative effect of social media usage was mediated by internet addiction, whereas when individuals use social media to build on preexisting offline social capital, their mental health is improved (Glaser et al., 2018). However, in the current study, I focused only on the problematic mobile social media usage among university students, using the problematic mobile social media usage assessment questionnaire; thus, we did not explore the positive effect of social media use on university students during the pandemic. Further studies should adopt multidimensional and comprehensive social media usage questionnaires to assess both the positive and negative effects of mobile social media usage on university students during the pandemic.

## Data Availability Statement

The raw data supporting the conclusions of this article will be made available by the authors, without undue reservation.

## Ethics Statement

The studies involving human participants were reviewed and approved by Tongji University. Written informed consent for participation was not required for this study in accordance with the national legislation and the institutional requirements.

## Author Contributions

The author confirms being the sole contributor of this work and has approved it for publication.

## Conflict of Interest

The author declares that the research was conducted in the absence of any commercial or financial relationships that could be construed as a potential conflict of interest.
